# Correction: Effects of small-sided games vs. simulated match training on physical performance of youth female handball players

**DOI:** 10.1371/journal.pone.0316740

**Published:** 2024-12-30

**Authors:** Rasa Mikalonytė, Rūtenis Paulauskas, Eduardo Abade, Bruno Figueira

There are errors in the author affiliations. The correct affiliations are as follows:

Rasa Mikalonytė^1^, Rūtenis Paulauskas^1^, Eduardo Abade^2,3^, Bruno Figueira^1,4^

**1** Educational Research Institute, Education Academy, Vytautas Magnus University, Vilnius, Lithuania, **2** Portugal Football School, Portuguese Football Federation, Oeiras, Portugal, **3** Research Center in Sports Sciences, Health Sciences and Human Development, CIDESD, University of Maia, ISMAI, Maia, Portugal, **4** Research Center in Sports Sciences, Health Sciences and Human Development, CIDESD, University of Tras os Montes e Alto Douro, UTAD, Vila Real, Portugal.

In the Author Contributions, Rūtenis Paulauskas should be listed as one of the persons who acquired financial support for the paper.
