# Supplementary material for: Effects of small-sided games vs. simulated match training on physical performance of youth female handball players
Source: PLoS One. 2022 Sep 13;17(9):e0273574. doi: 10.1371/journal.pone.0273574 (PMC9469977; doi:10.1371/journal.pone.0273574)
Supplement: Supplementary file 2 [file pone.0273574.s007.pdf]

ACCEPTED
