# Supplementary material for: Effects of small-sided games vs. simulated match training on physical performance of youth female handball players
Source: PLoS One. 2022 Sep 13;17(9):e0273574. doi: 10.1371/journal.pone.0273574 (PMC9469977; doi:10.1371/journal.pone.0273574)
Supplement: Attachment — Submitted filename: Review.pdf [file pone.0273574.s002.pdf]

Specific and general comments (by section)

ABSTRACT

- Set the eta square symbol appropriately
- Remove units of measurement from results
- Delete the subjective consideration ‘a greater extent’ from results

INTRODUCTION

- The introduction presents interesting content about small-sided games, such as the objectives pursued with their implementation, the results associated with handball or the advantages of their use. However, in the first paragraph, it would be useful to briefly introduce the concept itself. That is, what are the SSGs and what are their main characteristics. This first paragraph, as a whole, seems to me to be quite adequate. However, I would split it in two (line 55) to talk, in its second part (new paragraph 2), about the comparison between SSG and SMHT. Previously, in paragraph 1, the concept of SMHT should be introduced and developed as it has been done with SSGs.
- In relation to the previous point, the published scientific evidence on SSG and SMHT in handball should be incorporated in a third paragraph and, therefore, develop the idea already expressed in lines 80-86. Thus, it would be possible to check, for example, how the SSG would evaluate changes in physical performance in conditions without competitive anxiety or, on the contrary, whether or not the SMHT are effective on the physical performance of male and female handball players. Therefore, developing the link between SSG and physical performance in handball (HR, external load, etc.).
- With regard to the relationship between SSG - SMHT and game activity profile (2nd part of the ‘Introduction’ section), the information on the inclusion of LPS systems and their differentiation from GPS seems to me to be timely. However, I do not fully appreciate the relationship between what can be measured by this technology, the SSG/SMHT and the game activity profile. The question that any reader could ask would be: do SSGs, based on the information extracted from LPS systems, really enhance specific game activity profiles? It would therefore be necessary to link the last two aspects with the SSGs.

MATERIAL AND METHODS

Sample:

- Please add the average handball experience of the players (important to contextualise the effects of SSGs) and whether the players had previous contact with this type of training.
- Please justify the exclusion criteria for goalkeepers (line 95). And add the number of them.
- Could the authors define accurately the elite level of the players: national team, youth league (U-18) or other? And if they are part of a talent detection and development programme, please could be specified it?

Study design:

- In terms of study design and participants, were the two randomised groups performed in both teams? That is, was there a control group (SMTH) and an experimental group (SSG) in both team 1 and team 2, or, conversely, were all players in one team a control group and those in the other team an experimental group? This aspect seems to become clearer with the implementation of the pre-test and post-test (lines 118-120), but specify it for the whole intervention. And, the groups were counterbalanced besides randomized?
- Furthermore, in order to make your research more rigorous, please add the following reference to support the duration of the training programme and the frequency of sessions per week.
    - Hammami, A., Gabbett, T. J., Slimani, M., & Bouhlel, E. (2018). Does small-sided games training improve physical-fitness and specific skills for team sports? A systematic review with meta-analysis. Journal of Sports Medicine and Physical Fitness, 58(10), 1446-1455.

Methodology (I would call this section 'Procedures' and I would include all the sub-sections (training protocols, schedule, fitness test, etc.)

- Protocols – SSG. Reference no. 4 (line 138) concerning the size of the pitch should be deleted or replaced by another reference concerning handball. In the study by Hill-Hass (2009), which deals with football, it has little or nothing to do with the handball field dimensions.
- Protocols – SSG. Please define the dimensions of the goal area in the SSGs. It is important in relation to the distance to be covered by the players.

- Protocols – SSG**.** The following comment is a personal doubt as a handball player, coach and teacher: Do the authors think that the introduction of mini-goals without goalkeeper is a more effective modification in a SSG than playing with goalkeeper in an official goal (3mx2m)?
- Protocols. Could the authors explain or justify active rest in SSGs and yet passive recovery in SMHTs?
- Protocols. SMTH. Were the players chosen by the coach in the SSGs, as was the case in the SMTHs? If this action was carried out with the aim of equalising the technical-tactical level of the team-groups, it should also have been carried out in the SSGs.
- Fitness tests. For physical assessments, the inter-test coefficient of variation (CV) and an intra-class correlation coefficient (ICC) should be provided as informative indices of inter-test variability and consistency of observers' measurements, respectively. The authors provide it in all tests except YYIRTL1 and SJ. Please provide them in these tests as well.
- Fitness tests. For the measurement of jumping ability, why was the DJ used instead of the CMJ or unilateral CMJ?

RESULTS

- In the footnotes to the tables, make sure that you only add the abbreviations in order of appearance (left-right and top-bottom). The footnote to Table 4 is not complete. Do not include redundant information such as *'The value expressed as mean and standard deviation (SD) in both game-based training (SSG) and (SMHT) groups'*, but insert the abbreviation (X ± SD) in the table itself.
- Lines 254-256 can be summarised as 'No statistically significant differences were found in any component of the load'.
- Provide a suitable format of Table 4

DISCUSSION

- According to the objective of the study connected to the game activity profile, it would be appropriate to add in the first paragraph - summary of the ‘Discussion’ section the following sentence: ‘the game motion

characteristics were not influenced by either SSG or SMHT, except for those associated with sprinting at speeds above 5.21 $m \cdot s^{-1}$)."

- Throughout the manuscript, but especially in the 'Discussion' section, the statement relating the number of players and factors associated with internal load, such as RPE and HR (lines 280-282), raises my doubts. Firstly, because the results provided in Table 1 are merely descriptive (X ± SD) and have not been statistically analysed. And secondly, because only considering this descriptive information, for example, the HR decreased when the number of players per team was reduced from 4 to 3, contrary to this statement. However, the biggest drawback is the one discussed in point 1 of this comment. On the other hand, the total distance covered has not been analysed in terms of the type of small-sided game but in the comparison between SSGs and SMHTs. The distance is likely to be greater the fewer players there are, but it needs to be demonstrated. Therefore, the case of Belka et al. (2009) is not the case in this study.
- On the other hand, the total distance covered in the YYRTL1 test is analysed, being greater after the SMTH protocol than after the SSG protocol (Table 2). However, this difference in physical performance does not translate to the game activity profile as there is no difference ($p = 0.443$) between the total distance recorded in the SMHT and the SSG. And that should be the main aspect to discuss with regard to aerobic capacity in handball. That is, why is there a difference in the tests but not in the training? The idea of lines 310-313, more elaborated by comparing the previous results, would be the right one to develop this explanation.

GENERAL COMMENT: In this section it is not sufficiently clear why the following results have been obtained: (1) SSG enhance jumping ability and sprint speed; (2) SMHT favour the development of aerobic capacity (*CAUTION, in test not in relation to game activity profile).* For example, on the SSG point, in addition to reducing the number of players and the size of the pitch, it would not be possible to investigate the application of adapted rules (e.g. inclusion or not of the goalkeeper - Table 1) or the disappearance of playing positions? It is another matter whether the corresponding justifications for all this, which indeed focus on

the aspect of physical and conditional demands, are well justified (e.g. ‘These short-term high intensity actions may impose higher physiological loads and also allow stimuli for muscle power development’ lines 287-288). Furthermore, in the subheadings of the 'Discussion' section, in my opinion, I would not refer to the possible explanations, but rather to the main results: increased jumping power, increased speed and increased alactic anaerobic power. Therefore, a possible structure of this section would be, results → explanations related to handball → justification with data extracted from the present study. Consider this study to improve this section:

- Clemente, F. M., Afonso, J., & Sarmento, H. (2021). Small-sided games: An umbrella review of systematic reviews and meta-analyses. PLoS One, 16(2), e0247067.

PRACTICAL APPLICATIONS

- The practical applications are general conclusions of the study, but not a list of specific practical actions that coaches or physical trainers can implement in handball training. Practically, it is the same content as in paragraph 1 of the ‘Discussion’ section.

CONCLUSIONS

- The conclusion is a replication of the first paragraph of the ‘Discussion’ and ‘Practical Applications’ sections. This part of the manuscript should add value to the results found.

REFERENCES

- Please check the format of the scientific journals in the ‘References’ section. You may not use abbreviations for some journals (i.e.: J Strength Cond Res.) and the full name for others (i.e.: European journal of sport science.).

MINNOR COMENTS

- Line 119. Delete ‘to’
- Line 125. Add the symbol ‘ºC’
- Line 141. Delete ‘court size’
- Furthermore, check especially for any missing or incorrect spelling, capitalisation, etc.
