# Supplementary material for: Effects of small-sided games vs. simulated match training on physical performance of youth female handball players
Source: PLoS One. 2022 Sep 13;17(9):e0273574. doi: 10.1371/journal.pone.0273574 (PMC9469977; doi:10.1371/journal.pone.0273574)
Supplement: Attachment — Submitted filename: Review 2.pdf [file pone.0273574.s005.pdf]

Specific and general comments (by section)

INTRODUCTION

- In relation to the previous point, the published scientific evidence on SSG and SMHT in handball should be incorporated in a third paragraph and, therefore, develop the idea already expressed in lines 80-86. Thus, it would be possible to check, for example, how the SSG would evaluate changes in physical performance in conditions without competitive anxiety or, on the contrary, whether or not the SMHT are effective on the physical performance of male and female handball players. Therefore, developing the link between SSG and physical performance in handball (HR, external load, etc.).

  Reviewer#: Although the link to anxiety status has been removed, the most relevant scientific evidence in relation to SSGs has not been incorporated. As the authors state in lines 96-97, there are studies, albeit few, that develop the link between SSGs and physical performance (or other factors) in handball. For this reason, the 'Introduction' section cannot be considered complete until such information is included. Please add a paragraph to this effect.

- With regard to the relationship between SSG - SMHT and game activity profile (2nd part of the 'Introduction' section), the information on the inclusion of LPS systems and their differentiation from GPS seems to me to be timely. However, I do not fully appreciate the relationship between what can be measured by this technology, the SSG/SMHT and the game activity profile. The question that any reader could ask would be: do SSGs, based on the information extracted from LPS systems, really enhance specific game activity profiles? It would therefore be necessary to link the last two aspects with the SSGs.

  Reviewer#: done. Add the reference in line 84.

MATERIAL AND METHODS

Sample:

- Please add the average handball experience of the players (important to contextualise the effects of SSGs) and whether the players had previous contact with this type of training.

Reviewer#: Information on the background of the players regarding their participation in this type of training (SSGs) has not been included. It is important to know if there was a selection bias in the study sample in relation to experience.

Study design:

- In terms of study design and participants, were the two randomised groups performed in both teams? That is, was there a control group (SMTH) and an experimental group (SSG) in both team 1 and team 2, or, conversely, were all players in one team a control group and those in the other team an experimental group? This aspect seems to become clearer with the implementation of the pre-test and post-test (lines 118-120), but specify it for the whole intervention. And, the groups were counterbalanced besides randomized?

  Reviewer#: In my opinion, assigning each of the players to a group, control or experimental, should be a prior task that every researcher o technical staff member can perform. And even more so, with an affordable number of participants. If the authors have not defined this aspect, add the sample randomisation method for the formation of the experimental and control groups. On the other hand, I cannot find the information on the teams' balance.

Procedures

- Protocols – SSG. Please define the dimensions of the goal area in the SSGs. It is important in relation to the distance to be covered by the players.

  Reviewer#: The regulation dimensions of a handball area imply a length of approximately 16 metres from the end line. If, as the authors state, the dimensions of the area were maintained for the different game formats: (i) in the 3vs3 and 4vs4 format played on a 20x20m court the playing space was excessively reduced; (ii) and furthermore, in the 2vs2 format (20x10m) it was materially impossible to introduce a goal area of these dimensions.

- Fitness tests. For physical assessments, the inter-test coefficient of variation (CV) and an intra-class correlation coefficient (ICC) should be provided as informative indices of inter-test variability and consistency of observers' measurements, respectively. The authors provide it in all tests except YYIRTL1 and SJ. Please provide them in these tests as well.

Reviewer#: Both coefficients (CV and ICC), which are still not provided, should be included in the 'Method' section as the authors themselves do for the other fitness tests (e.g. lines 198-199 and 218-129).

RESULTS

- Lines 254-256 can be summarised as 'No statistically significant differences were found in any component of the load'.

  Reviewer#: Add '$p > 0.05$' at the end of the sentence. Remember it is a 'Result' section

DISCUSSION – GENERAL COMMENT:

Reviewer#: I am unable to find the differences between the 'Discussion' section of the original submission and this one in the second. While the changes have been made to the subtitles, I am unable to identify the changes made (please highlight the changes in red or activate the *tracked changes* function in your document to detect them. Also, add in the 'authors' response' the lines in the new manuscript where these changes have been made. Do this for the whole document). So, I copy my concerns again: In this section it is not sufficiently clear why the following results have been obtained: (1) SSG enhance jumping ability and sprint speed; (2) SMHT favour the development of aerobic capacity (*CAUTION, in test not in relation to game activity profile).* For example, on the SSG point, in addition to reducing the number of players and the size of the pitch, it would not be possible to investigate the application of adapted rules (e.g. inclusion or not of the goalkeeper - Table 1) or the disappearance of playing positions? It is another matter whether the corresponding justifications for all this, which indeed focus on the aspect of physical and conditional demands, are well justified (e.g. 'These short-term high intensity actions may impose higher physiological loads and also allow stimuli for muscle power development' lines 287-288). Finally, I consider that the reference mentioned (Clemente et al. 2021) is more than timely to vertebrate this section.

REFERENCES

- Modify the reference in line 350 according to journal style

MINNOR COMENTS

- If you want to use abbreviations in the ‘Conclusion’ section, please do so in all the terms included (line 367)
