# Supplementary material for: Effects of small-sided games vs. simulated match training on physical performance of youth female handball players
Source: PLoS One. 2022 Sep 13;17(9):e0273574. doi: 10.1371/journal.pone.0273574 (PMC9469977; doi:10.1371/journal.pone.0273574)
Supplement: Attachment — Submitted filename: General comments.docx [file pone.0273574.s003.docx]

**Effects of small-sided games vs. simulated match training on physical performance of youth female handball players**

**General comments to the authors**

Overall, this is a nice study that has some potential practical applications integrated with female soccer players during small-sided games in handball. The authors are commended on their efforts thus far. The study is well designed and well-written, with a great introduction proposing the usefulness of the topic and a clear outline of the research question. However, I suggest only small corrections and the authors should update the recent references about small-sided games. These corrections and studies will allow improving the manuscript.

**Abstract**

Instead of The results showed larger improvements in drop jump (cm)
(p=0.001, ηp2=0,219), jump power (w) (p=0.024, ηp2=0,232), absolute and relative
anaerobic alactic power (W and W·kg -1 ) (p=0.003, ηp2=0,248 and p=0.000,
ηp2=0,358 respectively) and 10 m sprint performance (p=0.000) in SSG group. SMHT
group improved Yo-Yo intermittent recovery test Level 1 distance (p=0.0001,
ηp2=0,368) to a greater extent

you should use this sentence clear and shortly

The results showed larger improvements in drop jump height, jump power, absolute and relative anaerobic alactic power and 10 m sprint performances following the SSG training compared with the SMHT (p ≤ 0.05, ηp2=ranging from 0.219 to 0.368).

Instead of drop jump (cm), you should use drop jump height.

Similarly performance responses, Game performance characteristics should be written.

**Introduction section**

Page 3, Line 48: The authors should add recent references about small-sided games

Arslan, E., Kilit, B., Clemente, F. M., Soylu, Y., Sögüt, M., Badicu, G., ... & Murawska-Ciałowicz, E. (2021). The Effects of Exercise Order on the Psychophysiological Responses, Physical and Technical Performances of Young Soccer Players: Combined Small-Sided Games and High-Intensity Interval Training. *Biology*, *10*(11), 1180.

Page 3, Line 50: The authors should add recent references about small-sided games

Arslan, E.; Kilit, B.; Clemente, F.M.; Murawska-Ciałowicz, E.; Soylu, Y.; Sogut, M.; Akca, F.; Gokkaya, M.; Silva, A.F. Effects of Small-Sided Games Training versus High-Intensity Interval Training Approaches in Young Basketball Players. Int. J. Environ. Res. Public Health **2022**, 19, 2931. https://doi.org/10.3390/ijerph19052931

Page 3 and 4: The authors should add recent references about small-sided games in handball and also discussion section

Jurišić, M. V., Jakšić, D., Trajković, N., Rakonjac, D., Peulić, J., & Obradović, J. (2021). Effects of small-sided games and high-intensity interval training on physical performance in young female handball players. *Biology of Sport*, *38*(3), 359.

**Methodology**

Page 5, Line 117: it should be (e.g., technical, tactical and strength)

Page 6, Line 119: it should be within each group (SSGs and SMHT), players were divided into

Page 6, Line 125: it should be (19–22 °C)

**Protocols**

Page 6, Line 135: which one is the your style you have to make a decision

10-min or 33 min. Please be careful throughtout the article

Page 6, Line 137: Instead of small-sided games, it should be SSG

Page 7, Line 146: Instead of rate of perceived exertion, it should be rating of perceived exertion

**SMHT**

Page 7, Line 157: Instead of to indicate their rate of perceived exertion (RPE), it should be to indicate their RPE

**Fitness identification**

Page 7, Line 164: you do not need (W)

Page 9, Line 200: Margaria-Kalamen AAP Test ??? what are they AAP???

**Results section**

This section is well designed and well-written

**Discussion section**

This section is well designed and well-written. However, the authors should add limitations and strengths of their article.

**Tables**

This section is well designed and well-shown
